# Comparison of the Frequency of Functional SH3 Domains with Different Limited Sets of Amino Acids Using mRNA Display

**DOI:** 10.1371/journal.pone.0018034

**Published:** 2011-03-21

**Authors:** Junko Tanaka, Hiroshi Yanagawa, Nobuhide Doi

**Affiliations:** Department of Biosciences and Informatics, Keio University, Yokohama, Japan; Cardiff University, United Kingdom

## Abstract

Although modern proteins consist of 20 different amino acids, it has been proposed that primordial proteins consisted of a small set of amino acids, and additional amino acids have gradually been recruited into the genetic code. This hypothesis has recently been supported by comparative genome sequence analysis, but no direct experimental approach has been reported. Here, we utilized a novel experimental approach to test a hypothesis that native-like globular proteins might be easily simplified by a set of putative primitive amino acids with retention of its structure and function than by a set of putative new amino acids. We performed *in vitro* selection of a functional SH3 domain as a model from partially randomized libraries with different sets of amino acids using mRNA display. Consequently, a library rich in putative primitive amino acids included a larger number of functional SH3 sequences than a library rich in putative new amino acids. Further, the functional SH3 sequences were enriched from the primitive library slightly earlier than from a randomized library with the full set of amino acids, while the function and structure of the selected SH3 proteins with the primitive alphabet were comparable with those from the 20 amino acid alphabet. Application of this approach to various combinations of codons in protein sequences may be useful not only for clarifying the precise order of the amino acid expansion in the early stages of protein evolution but also for efficiently creating novel functional proteins in the laboratory.

## Introduction

Although modern proteins usually consist of 20 different amino acids, it has been proposed that amino acid members in primitive proteins varied during the early stage of protein evolution [1]–[6]. It has been inferred that the primordial genetic code was composed of a smaller set of amino acids because prebiotic synthesis on the primitive earth is thought to have been inadequate for 20 different amino acids [1]. In the coevolution hypothesis, it is proposed that the genetic code coevolved with the amino acid biosynthetic pathways, and additional amino acids were introduced after production through their synthetic pathways [4]. Comparative genome sequence analysis of orthologous proteins in the genomes of bacteria, archaea and eukaryota revealed that the frequencies of Gly, Ala, Glu and Pro in proteins consistently decrease (*i.e*., primitive amino acids that are assumed to have been the first incorporated into the genetic code), while the frequencies of Ser, His, Cys, Met and Phe increase (*i.e*., new amino acids that are assumed to have recently been added to the genetic code) over the course of protein evolution [5]. The trend of amino acid gain and loss is in agreement with the likely order of incorporation of amino acids into the genetic code, as deduced from other criteria [3].

Several protein design experiments have proved that the full set of 20 amino acids is not necessarily essential for protein structure and function [7]–[14]. For example, Riddle *et al*. generated simplified SH3 domains (a small β-sheet protein) from a combinatorial library that was composed of five different amino acids by using a phage display technique [8]. Further, Hecht’s group created four helix bundle proteins with 11 amino acids [7], [12], [13], and Jumawid *et al*. generated α3β3 *de novo* proteins with seven amino acids [14]. However, these experiments have attempted to generate simplified proteins with fewer amino acids than the natural proteins, and they have not focused on whether the accepted amino acids are primitive or not. Previously, Babajide *et al*. demonstrated *in silico* that native-like folded structures of several tested proteins are maintained with a restricted alphabet mainly containing primitive amino acids (Ala, Gly, Leu and Asp) but were not maintained with a set of nonprimitive amino acids (Gln, Leu and Arg) [15]. To test this hypothesis experimentally, we sought to compare the function and structure of tested proteins with different subsets of amino acids for the first time.

**Figure 1:**
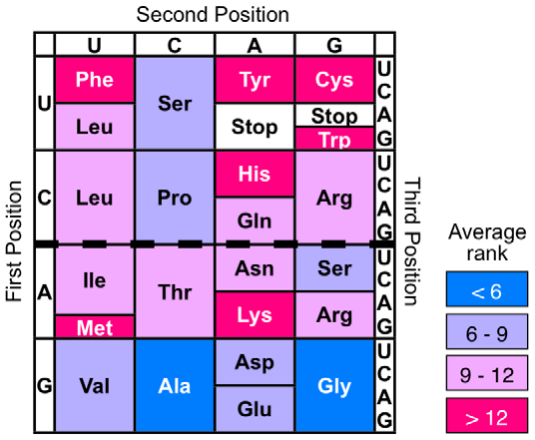
The universal genetic code. The average rank represents the chronological order of amino acid addition to the genetic code. The values calculated from ∼60 criteria were Gly, 3.5; Ala, 4.0; Asp, 6.0; Val 6.3; Pro, 7.3; Ser, 7.6; Glu, 8.1; Thr, 9.4; Leu, 9.9; Arg, 11.0; Asn, 11.3; Ile, 11.4; Gln, 11.4; His, 13.0; Lys, 13.3; Cys, 13.8; Phe, 14.2; Tyr, 15.2; Met, 15.4; Trp, 16.5 (data from [3]). The upper half of the table of the genetic code corresponding to codon YNN (Y  =  T or C; N  =  T, C, A or G) contains a lot of newly added amino acids (*e.g*., Phe and Cys), while the lower half corresponding to codon RNN (R  =  A or G) contains the most primitive amino acids, Ala and Gly.

As a first attempt, we designed randomized *src* SH3 gene libraries in which approximately half the residues of the SH3 gene were replaced by randomized codons in the lower or upper half of the table of the genetic code (Fig. 1). The SH3 domain is one of the most common mediators in intracellular signaling pathways. Because the SH3 domain is a well-known protein and thus the conserved positions that play important roles in structure and function have already been examined [16], we can randomize only non-conserved regions. A subset of amino acids that are coded by the lower half of the genetic code are mainly putative primitive amino acids (*e.g*., Ala and Gly), whereas a subset of amino acids that are coded by the upper half contains many putative new amino acids (*e.g*., Cys, Phe, Tyr and Trp).

From these randomized libraries, functional SH3 sequences were selected using mRNA display [17], [18]. In mRNA display, each cell-free translated polypeptide in a library covalently binds to its corresponding mRNA through puromycin. After affinity selection *via* the protein portion of an mRNA-displayed protein library, selected proteins can be easily identified by amplification and sequencing of the mRNA portion. Moreover, mRNA display based on cell-free translation can handle larger number of molecules (approximately 10^12–13^) than the other cell-based display technique such as phage display, and it makes possible enrichment of active sequences with low abundance from a library with high diversity and complexity. Therefore, we used mRNA display to elucidate and compare the frequency of functional SH3 sequences in randomized SH3 libraries with different sets of amino acids.

## Results

### Design and construction of randomized SH3 libraries

**Figure 2:**
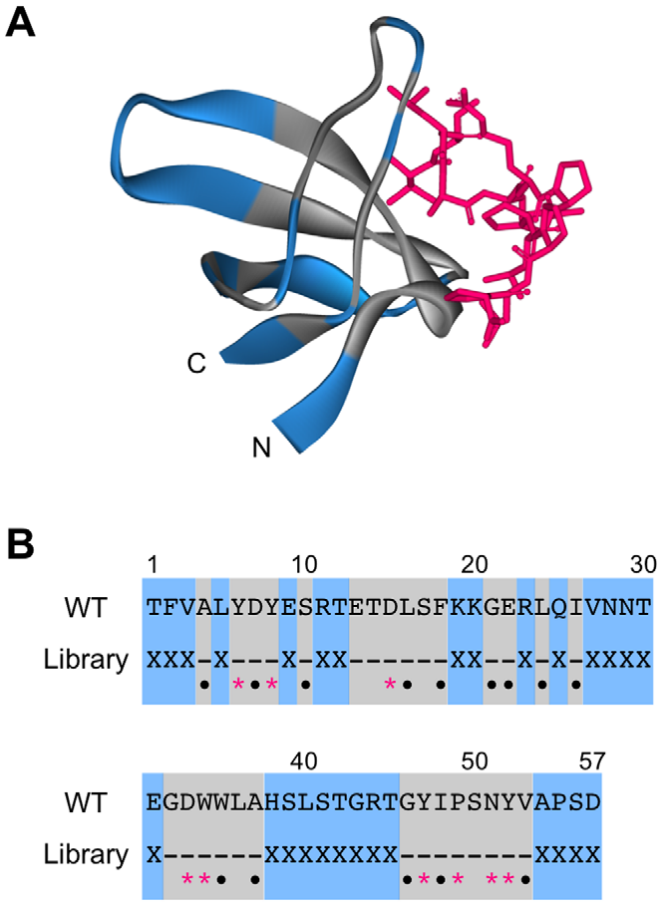
The three-dimensional structure and sequence of the *src* SH3 domain. (A) The three-dimensional structure of the complex of the SH3 domain with the VSL12 peptide. The randomized and conserved positions of the SH3 domain in this study are shown in blue and gray, respectively. The peptide ligand VSL12 is shown in red. Structure was visualized by Accelrys DiscoveryStudio 2.1 (PDBid: 1QWF, [21]). (B) The amino-acid sequence of the SH3 domain. The randomized amino acids (X) are shown in blue. In the highly conserved region (gray), red asterisks indicate residues contacting the peptide ligand and black dots indicate residues that are important in determing the domain structure [8], [16].

First, we constructed partially (28 out of 57 amino acids) randomized SH3 gene libraries, SH3(RNN)_28_ and SH3(YNN)_28_, with randomized codons RNN (R  =  A or G; N  =  T, C, A or G) and YNN (Y  =  T or C), corresponding to the lower and upper half of the table of the genetic code, respectively (Fig. 1). We also prepared a randomized SH3 gene library SH3(NNN)_28_ with all 20 amino acids as a control. If particular amino acid residues are essential for a randomized position of the SH3 gene, the frequency of occurrence of functional proteins will be greatly affected. To exclude this possibility, the randomized codons were introduced into 28 out of 57 amino acid residues of the *src* SH3 domain and not in the highly conserved residues of the SH3 domain (Fig. 2), such as the ligand peptide binding region, the hydrophobic core region and the surface region, that prefers polar amino acids [16]. Furthermore, to match the biophysical properties (the proportion of hydrophobic residue and the tendency to form β-sheet) of randomized SH3 proteins among three libraries, the mixed-base compositions of random regions (N, R and Y) were designed to provide amino acid compositions resembling modern proteins.

The random regions of the SH3(NNN)_28_ and SH3(YNN)_28_ libraries contain stop codons. In addition, when the randomized DNA was cloned and sequenced, more than 90% of sequences in each library contained unexpected frameshifts (data not shown), probably due to errors during chemical synthesis. Thus, we sought to eliminate sequences containing stop codons and frameshifts by preselection using mRNA display, as described previously [19], [20]. In mRNA display, the C-terminus of the *in vitro* translated polypeptide is covalently bound to the 3' terminus of the template mRNA without stop codons on the stalling ribosome. Thus, mRNA sequences with stop codons cannot form mRNA-displayed proteins. Further, the C-terminal FLAG tag encoded by mRNA sequences with frameshifts cannot be properly translated. Thus, these mRNA sequences with stop codons or frameshifts are principally removed from libraries by purification with anti-FLAG antibody-immobilized beads. Indeed, the percentages of sequences without stop codons or frameshifts in the SH3(RNN)_28_, SH3(YNN)_28_ and SH3(NNN)_28_ libraries increased from 8, 2 and 6% to 72, 25 and 33%, respectively, after one round of preselection.

### Selection of functional sequences from the randomized SH3 libraries

**Figure 3:**
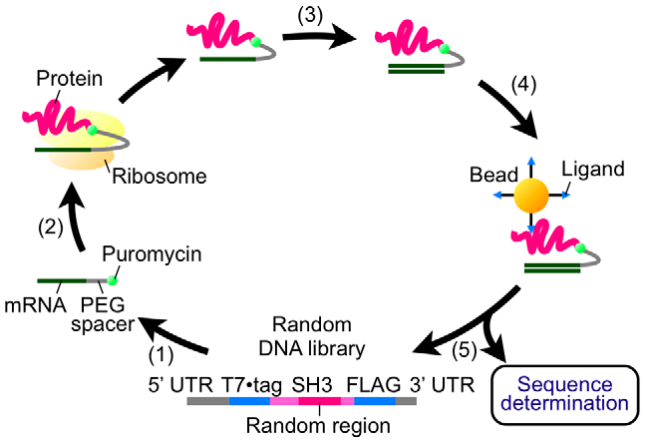
Schematic representation of mRNA-display selection of functional SH3 sequences. (1) A DNA library encoding a randomized SH3 domain with an N-terminal T7·tag and a C-terminal FLAG tag was transcribed and ligated with a PEG-puromycin spacer. (2) The modified mRNA library was translated *in vitro*, and (3) the resulting mRNA-protein conjugates were purified with anti-FLAG antibody-immobilized beads and reverse-transcribed. (4) The mRNA/DNA-protein conjugates were incubated with the ligand peptide-immobilized beads, washed and competitively eluted with the free ligand peptides. (5) The DNA portion of the eluted molecules was amplified by PCR to form a randomized DNA library for the next round.

**Figure 4:**
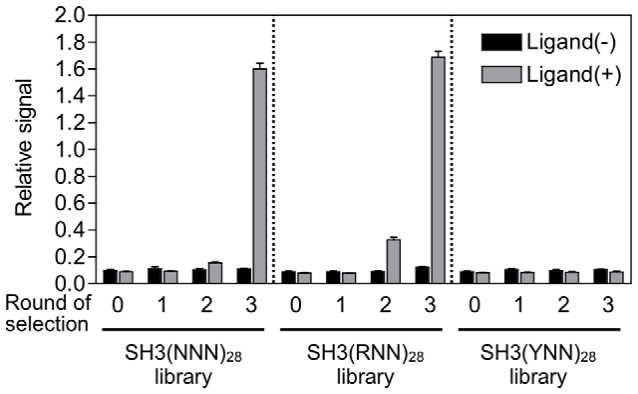
Fraction of functional SH3 sequences at each round of selection. The total amount of three kinds of libraries, SH3(NNN)_28_, SH3(RNN)_28_ and SH3(YNN)_28_, that bound to the peptide ligand before (0) and after 1–3 rounds of selection were quantified by ELISA (gray bars). Black bars show negative controls (no ligand peptide was immobilized). Error bars indicate s.d. of four samples.

*In vitro* selection of functional SH3 sequences that can bind to the ligand peptide VSL12 [21] from each library (containing 3×10^13^ molecules with up to 0.6–2.4×10^11^ diversity) was performed by mRNA display (Fig. 3). The procedure was the same as that of the preselection except for the following two points: (i) the mRNA portion of the mRNA-displayed protein was reverse-transcribed to form an RNA/DNA hybrid to prevent binding of RNA with a particular secondary structure, and (ii) the mRNA/DNA-displayed proteins were selected with VSL12-immobilized beads. After three rounds of selection, DNAs were amplified by polymerase chain reaction (PCR), translated *en masse* and analyzed by enzyme-linked immunosorbent assay (ELISA). Consequently, the fraction of functional SH3 sequences capable of binding to the VSL12 peptide increased in the SH3(RNN)_28_ library after 3 rounds of selection (Fig. 4), but not in the SH3(YNN)_28_ library after even 5 rounds of selection (Fig. S1).

**Figure 5:**
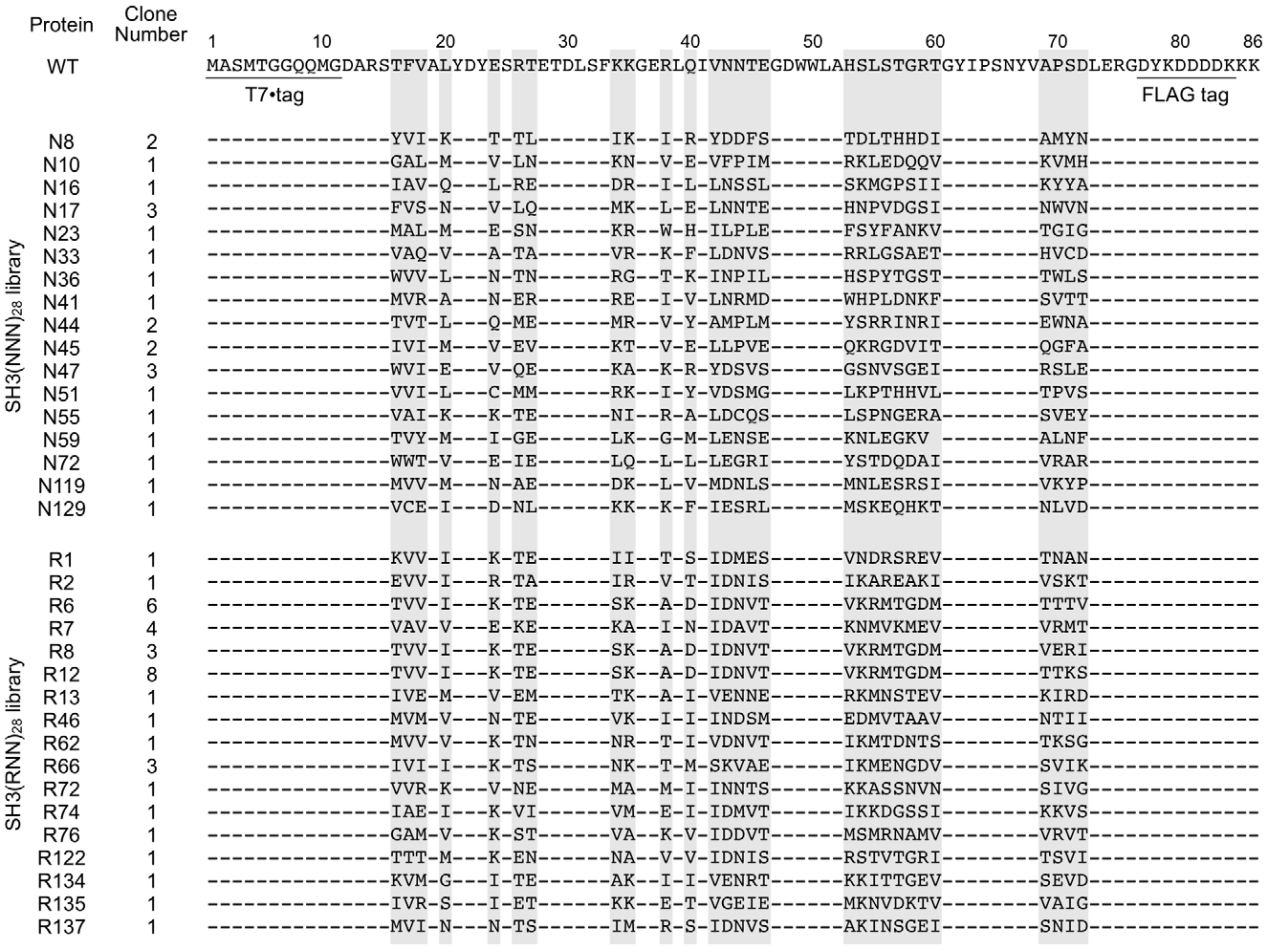
Amino acid sequences of selected proteins. Amino acid sequences of the full-length of wild-type (WT) SH3 domain and the randomized region (highlighted in gray) of selected proteins from the SH3(NNN)_28_ and SH3(RNN)_28_ libraries are shown. Each sequence contains the N-terminal T7·tag (1–11 aa) and the C-terminal FLAG tag (77–84 aa). Dashes indicate amino acids that are identical with WT.

Furthermore, the binding signal appeared in the 2nd round of the SH3(RNN)_28_ library but not in that of the SH3(NNN)_28_ library (Fig. 4). The selected DNAs from the 3rd round of SH3(RNN)_28_ and SH3(NNN)_28_ libraries were cloned, and over 90 randomly chosen clones from each library were sequenced. Because more than half of selected sequences contained frameshifts, we obtained 36 and 24 sequences without frameshift from SH3(RNN)_28_ and SH3(NNN)_28_ libraries, respectively (Fig. 5). All clones with no frameshift bound to VSL12 peptide by ELISA (see next section). The random regions of the selected amino acid sequences shared low-sequence similarity with the wild-type sequence (0% to 29% identical). Further, alignment of the selected sequences indicated that approximately half of the 3rd round of the SH3(RNN)_28_ library was dominated by the closely related sequences (R6, R8 and R12) and differed by only four residues (69–72 aa) (Fig. 5), suggesting that they were derived from a single ancestral sequence. On the other hand, the functional SH3 sequences from the 3rd round of the SH3(NNN)_28_ library have no such closely related sequences (Fig. 5). These results suggest that the functional SH3(RNN)_28_ sequences were enriched earlier than the functional SH3(NNN)_28_ sequences, and it presumably caused recombination between the formerly enriched sequences in the SH3(RNN)_28_ library during PCR.

Next, we characterized the function and structure of the selected proteins arbitrarily chosen from the 3rd round of the SH3(RNN)_28_ and SH3(NNN)_28_ proteins to test whether the biophysical properties of the SH3(RNN)_28_ and SH3(NNN)_28_ proteins were similar.

### Binding properties of selected proteins

**Figure 6:**
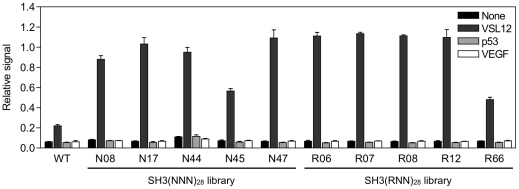
Binding specificity of selected proteins. Relative binding of selected proteins from the SH3(NNN)_28_ and SH3(RNN)_28_ libraries to several kinds of peptide (black, none; dark gray, VSL12; light gray, p53_371–380_; white, VEGF_84–91_) was analyzed by ELISA. WT, wild-type SH3 domain. Error bars indicate s.d. of four samples.

First, we characterized the binding specificity of 10 plurally-obtained clones (N8, N17, N44, N45, N47, R6, R7, R8, R12 and R66; Fig. 5) from the 3rd round of the SH3(RNN)_28_ and SH3(NNN)_28_ libraries by ELISA. Consequently, all selected proteins bound to the VSL12 peptide but did not bind to other nonrelated peptides (Fig. 6).

Next, we characterized the binding affinity of purified proteins to VSL12 peptide by fluorescence perturbation assays. Eight SH3(RNN)_28_ and seven SH3(NNN)_28_ proteins were overexpressed in *E. coli* and purified by using the C-terminal His_6_-tag (Fig. S2) under denaturing condition because of their low solubility. Although we obtained two soluble proteins from the RNN library (data not shown), we did not use the soluble fraction for further characterization because of their low expression level. After refolding of denatured purified proteins, three SH3(RNN)_28_ proteins (R1, R12 and R13) and two SH3(NNN)_28_ proteins (N17 and N47) as well as the wild-type SH3 domain were obtained without aggregation. The affinities of the SH3(RNN)_28_ and SH3(NNN)_28_ proteins were similar to each other (0.44–0.74 µM) and 3- to 5-fold higher than the wild-type SH3 domain (Table 1).

**Table 1:** Kinetic and thermodynamic parameters for the wild-type and SH3 variants.

Protein	*K*_d_ (µM)	*T*_m_ (K)	*ΔH*_m_ (kJ/mol)
Wild-type	2.30±0.47	345.2±0.5	195.9±13.5
N17	0.55±0.07	312.9±0.7	122.8±9.0
N47	0.53±0.09	306.6±1.8	88.7±10.6
R1	0.70±0.11	304.5±1.6	92.6±9.4
R12	0.74±0.13	309.7±6.2	73.4±23.9
R13	0.44±0.07	330.8±0.5	123.8±6.0

### Structural characterization of selected proteins

**Figure 7:**
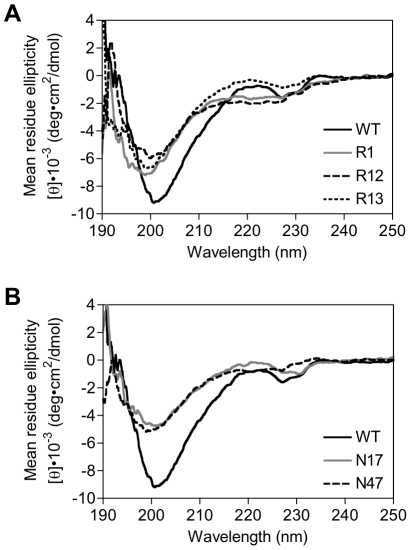
Circular dichroism spectra of selected proteins. (A) The mean residue ellipticity values of wild-type SH3 domain (black solid line) and SH3 variants R1 (gray solid line), R12 (dashed line) and R13 (dotted line) selected from the SH3(RNN)_28_ library are shown. (B) The mean residue ellipticity values of wild-type SH3 domain (black solid line) and SH3 variants N17 (gray solid line) and N47 (dashed line) selected from the SH3(NNN)_28_ library are shown.

We analyzed the secondary structure of the purified proteins R1, R12, R13, N17 and N47 by means of circular dichroism (CD) spectroscopy. Although the CD spectra of β-sheet proteins usually have minima at ∼217 nm, native SH3 domain has unusual maxima at 220 nm that may be a result of the environment of aromatic residues or β-turn conformations [22]. Our results showed that all SH3 domain variants, especially R13 and N17, had the typical maxima at 220 nm for the wild-type SH3 domain (Fig. 7 and Fig. S3), though the peak intensities were varied, suggesting that they have similar secondary structure to the wild-type.

**Figure 8:**
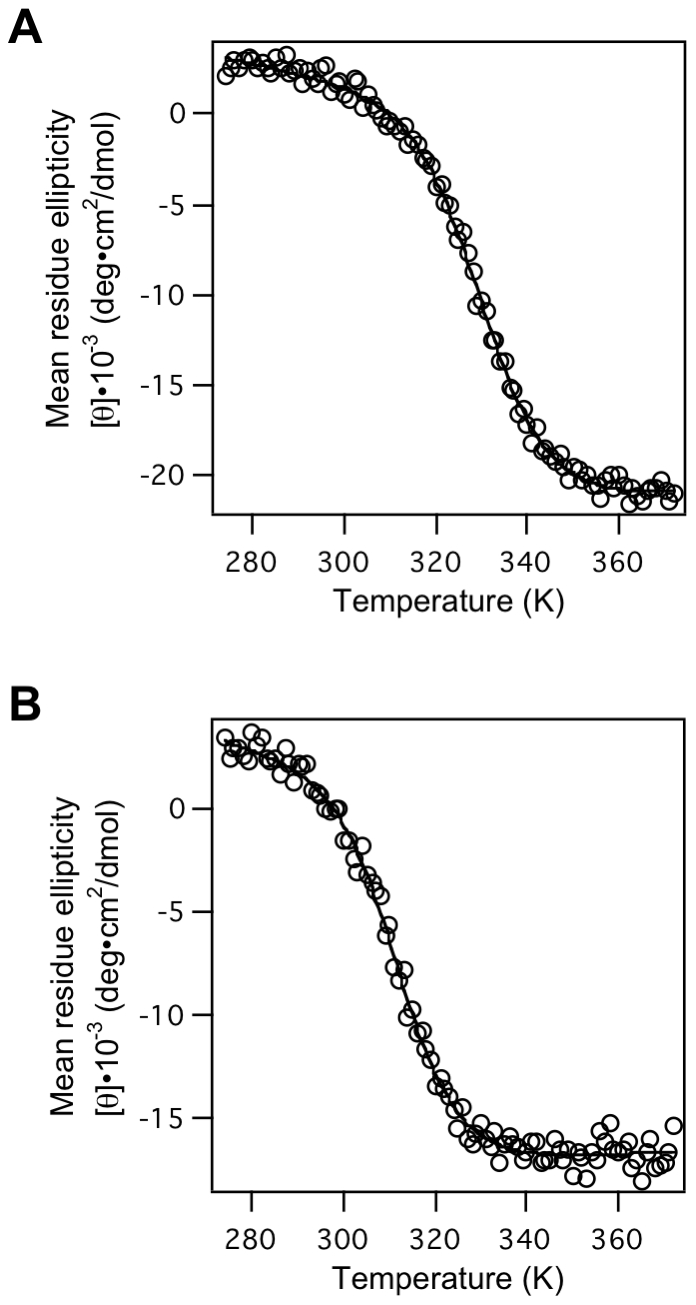
Thermal unfolding curves of selected proteins. The CD mean residue ellipticity values at 220 nm for (A) R13 and (B) N17 were monitored as a function of temperature. The solid curves are the best fit of the whole set of data to eq. 1 (Materials and Methods).

The thermal stabilities of the selected proteins were estimated from the thermal denaturation curves of the CD value at 220 nm. They exhibited two-state cooperative thermal unfolding (Fig. 8), and the denaturation processes were reversible. Though all variants were less stable than the wild-type SH3 domain, they showed a wide range (*ΔH*_m_ values, 73.4 kJ/mol to 123.8 kJ/mol) of thermodynamic stabilities (Table 1).

These results indicated that the secondary structures and thermal stabilities of proteins selected from both the SH3(RNN)_28_ and SH3(NNN)_28_ libraries were similar to each other but lower than those of wild-type, in spite of their native-like specificity and higher affinity for the SH3 ligand peptide. These results are in contrast with those from a previous study in which SH3 variants with a simplified alphabet revealed a lower affinity and higher stability than the wild-type [8]. One of the reasons could be the difference in selection method, as we used mRNA display instead of phage display. Phage display is a multivalent display technique, and thus not only high-affinity binders but also low-affinity binders are captured by avidity effects, while mRNA display is a monovalent display technique. In addition, since phage-displayed proteins are expressed in *E. coli*, unstable SH3 variants might tend to be degraded by proteases or aggregated in *E. coli*, and thus only stable proteins may be selected in the previous study.

## Discussion

In this study, functional SH3 sequences were enriched from a SH3(RNN)_28_ library but not from a SH3(YNN)_28_ library even after additional 2 rounds of selection. We roughly estimated that the SH3(RNN)_28_ library includes >10,000-fold larger number of functional SH3 sequences than the SH3(YNN)_28_ library because the enrichment efficiency was 200–3,000-fold per round calculated from the abundance in each round of two selected clones from each library by using real-time PCR (See Materials and Methods). We predicted that this would not be explained by the differences in typical biophysical properties (*e.g*., charge and hydrophobicity) of individual amino acids coded by RNN with those by YNN because we reconstructed the randomized SH3 domain in which highly conserved positions were fixed. If negatively charged amino acids (Glu and Asp) in a position of the randomized region of SH3 domain are essential for the SH3 activity, no functional sequence will be obtained from the SH3(YNN)_28_ library because YNN codes no negatively charged amino acids. However, negatively charged amino acids in the randomized region would not be essential, because the number of Glu and Asp in the region was zero in a selected active clone N36 (Fig. 4). Further, the percentage of hydrophobic residues (Ala, Val, Leu, Ile, Met, Phe, Trp and Tyr) in the initial SH3(YNN)_28_ library (43%) is almost equal to that of the 3rd round of the SH3(RNN)_28_ library (42%) as well as the SH3(NNN)_28_ library (44%) (Table S1).

Our result experimentally supports the Babajide's hypothesis [15], for the first time, that modern proteins might be able to be simplified by a set of putative primitive amino acids more easily than by a set of putative new amino acids. The reason is still unknown but may reflect an evolutionary constraint that primordial proteins consisted of a small set of primitive amino acids and gradually acquired new amino acids in the course of neutral evolution. To strengthen this hypothesis, not only a β-sheet protein used in this study but also an α-helical protein and other protein folds should be tested. Moreover, application of this approach to various combinations of codons in a protein sequence may be useful for clarifying the precise order of the amino acid expansion in the early stages of protein evolution.

Further, interestingly, the functional SH3 sequences were enriched from the SH3(RNN)_28_ library slightly earlier than from the SH3(NNN)_28_ library, while the function and structure of selected SH3(RNN)_28_ proteins with the primitive alphabet were comparable with those of SH3 domains with the 20 alphabet. The results imply that the protein sequence variety with a limited set of primitive amino acids includes a larger number of functional sequences than that with the current 20 amino acid alphabet. Previously, it has been reported that such reduced alphabets are effective for functional selection from randomized libraries [23], [24]. However, in these studies, only a few amino acids in the active sites were randomized. In this study, we showed that a limited set of primitive amino acids are also effective for wide frame regions, excluding the active sites.

In future work, it would be extremely interesting to randomize both active sites and frame regions and to examine whether the resulting wholly random-sequence library with limited alphabets is suited for *in vitro* selection of functional sequences, as the occurrence rate of functional sequences in a random-sequence library with a natural 20 alphabet has been shown to be quite low [25]. In our previous study, the random-sequence proteins with primitive alphabets tended to be more soluble as compared to random-sequence proteins with the natural alphabet [20], [26]. Similarly, in this study, 2 of 8 functional proteins from the SH3(RNN)_28_ library were expressed in the soluble fraction in *E. coli*, while none of the 7 functional proteins from the SH3(NNN)_28_ library were expressed in the soluble fraction (data not shown). Thus, the design of proteins with a higher content of primitive amino acids may improve the solubility as well as the rate of folded and functional proteins. Again, various subsets of amino acids including putative primitive amino acids should be tested for functional selection depending on the target function, because some putative new amino acids may be essential for some function. For example, His and Cys are essential residues for binding to zinc ions in the zinc-finger motif, and Cys is required for stabilization of extracellular domains by disulfide bonds. Combining of putative primitive amino acids and some particular new amino acids depending on the target protein would provide attractive resource for design and evolution of novel proteins in the laboratory.

## Materials and Methods

### Construction of randomized DNA libraries

All oligonucleotides used in this study were synthesized by Sigma-Aldrich, Japan (Table S2). Each of three randomized libraries, SH3(RNN)_28_, SH3(YNN)_28_ and SH3(NNN)_28_, was constructed by overlap-extension PCR from an equimolar mixture (8 pmol each) of four DNAs (Fragment 1–4; Tables S2 and S3) containing a random sequence region flanked by constant sequences using the primers SPO7tagF-mut2 and FLAG1A-mut2 (Table S2). The PCR products were purified with a QIAquick PCR purification kit (Qiagen).

### Preselection of randomized DNA libraries using mRNA display

Preselection by mRNA display was performed as previously described [19], [20]. Briefly, the purified DNA (∼5 pmol, 3×10^12^ molecules) was transcribed with a RiboMax large-scale RNA production system-SP6 (Promega). The resulting RNA was purified with an RNeasy mini kit (Qiagen) and ligated with polyethylene glycol (PEG)-puromycin spacer [p(dCp)_2_-T(Fluor)p-PEGp-(dCp)_2_-puromycin] using T4 RNA ligase (Takara). The ligated RNA was purified with the RNeasy mini kit and translated with wheat germ extract plus (Promega) for 1 h at 25°C. The reaction mixture containing mRNA-displayed proteins (6×10^13^ molecules with 3×10^12^ potentially different sequences) was added to anti-FLAG M2 antibody-immobilized agarose beads (Sigma-Aldrich) and mixed on a rotator for 1 h at 4°C. The beads were washed with 500 µl of TBST (Tris-buffered saline with 0.2% Tween 20, pH 7.4) four times. The mRNA-displayed proteins were eluted with TBST containing 1 mg/ml FLAG M2 peptide (Sigma-Aldrich) at 4°C for 1 h. The mRNA portion of the eluted mRNA-displayed proteins was amplified by reverse-transcription (RT)-PCR with a OneStep RT-PCR kit (Qiagen) using the primers SPO7tagF-mut2 and FLAG1A-mut2. The RT-PCR products were purified with the QIAquick PCR purification kit and were served as randomized DNA libraries for further functional selection.

### *In vitro* selection of functional SH3 sequences using mRNA display

From the above DNA libraries, the mRNA-displayed protein libraries were generated as described above, mixed with the anti-FLAG M2 antibody-immobilized agarose beads again, and washed with 300 µl of TBST three times. Then the RT reaction mixture with Superscript II (Invitrogen) and FLAG M2 peptide were added and incubated for 1 h at 37°C to form the RNA/DNA hybrid. The resulted mRNA/DNA-displayed protein libraries were exchanged into TBST on Bio-gel P-30 (BioRad) gel filtration columns and then incubated for 1 min at 4°C with Streptavidin coating Magnotex-SA beads (Takara) preblocked with DIG blocking Buffer (Roche), salmon sperm DNA (Stratagene) and yeast RNA (Sigma-Aldrich) to avoid non-specific binders. The supernatants were incubated with biotinylated SH3 peptide ligand VSL12 (Invitrogen; Biotin-XXXVSLARRPLPPLP, X  =  Aminohexanoic acid) for 1 h at 4°C, and the complexes of mRNA/DNA-displayed proteins and the biotinylated peptides were captured on Magnotex-SA beads for 1 min at 4°C. After washing with 300 µl of TBST three times, the bound mRNA/DNA-displayed proteins were eluted with TBST containing 1 mM free VSL12 peptide (Invitrogen; VSLARRPLPPLP) for 5 min at 4°C. The eluate of each library was used for PCR amplification with the primers SPO7tagF-mut2 and FLAG1A-mut2. The resulted DNA was purified, and served as template DNA for next round of selection or cloned using a TOPO TA cloning kit (Invitrogen) followed by sequencing with an ABI PRISM 3100 genetic analyzer (Applied Biosystems).

### Real-time PCR analysis

Real-time PCR was performed with SYBR *Premix Ex Taq* II (Takara) using protein-specific primer sets, N8-F and N8-R, N16-F and N16-R, R2-F and R2-R, R6-F and R6-R (Table S2) on the LightCycler (Roche).

### Enzyme-linked immunosorbent assay (ELISA)

Streptavidin transparent C8 plates (Nunc) were incubated with 1 µM biotinylated peptide [VSL12, p53_371–380_ (Invitrogen; Biotin-SKKGQSYSRH), and VEGF_84–91_ (Invitrogen; Biotin-XXPHQGQHIG, X  =  Aminohexanoic acid)] for 1 h at 25°C, and washed with TBST. The RNA libraries from each round of the selection or the RNA of selected clones were translated using wheat germ extract (Promega) for 2 h at 25°C. The translated product was transferred into wells of the above plate with or without immobilized peptide and then incubated for 1 h at 25°C. After washing with TBST, the plate was incubated with HRP-conjugated anti-FLAG M2 antibody (Sigma-Aldrich) for 1 h at 25°C. After washing, the amounts of bound molecules of each library or the selected clones were detected by using TMB substrate kit (Nacalai Tesque). The absorbance at 450 nm (reference wavelength at 655 nm) was measured with a microplate reader (Safire, Tecan).

### Cloning, overexpression and purification of selected proteins

The random regions of the clones were digested with BglII and XhoI and subcloned into the pET20 vector (Novagen) containing the N-terminal T7·tag sequence and the C-terminal His_6_ tag sequence. The individual plasmids were transformed into *Escherichia coli* BL21(DE3)-CodonPlus cells (Stratagene). The bacteria were grown in LB broth containing 100 µg/ml ampicillin and 34 µg/ml chloramphenicol at 37°C, and protein expression was induced by adding 0.5 mM isopropyl-β-D-thiogalactopyranoside. After an additional 5 h of growth, the bacteria were harvested by centrifugation and lysed in a BugBuster (Novagen) containing a protease inhibitor cocktail (Sigma-Aldrich). The centrifuged supernatants were used as soluble fractions. The pellets were resuspended in a buffer containing 8 M urea, and the supernatants after centrifugation were used as insoluble fractions. The proteins of selected clones were purified by affinity chromatography under denaturing condition using Ni-NTA Superflow resin (Qiagen), from which they were eluted with a pH gradient under denaturing condition. The purified denatured proteins were dialyzed against 50 mM phosphate buffer (pH 7.4) for refolding. The soluble and insoluble fractions and purified proteins were separated by 16.5% Tricine sodium dodecyl sulfate-polyacrylamide gel electrophoresis (SDS-PAGE) and detected by Coomassie brilliant blue staining. The protein concentrations were determined using a BCA protein assay kit (Pierce).

### Circular dichroism (CD) measurements

CD measurements were performed with a J-820 spectropolarimeter (Jasco). CD spectra of purified proteins (10 µM) were measured from 190 to 250 nm at 20°C using a 2 mm path-length cell. The results were expressed as mean residue molar ellipticity [*θ*]. Thermal denaturation was monitored by following the change in ellipticity at 220 nm using a 10 mm path-length cell. The temperature was increased at 2°C/min. The reversibility of thermal denaturation was tested by stepwise cooling of the protein solution back to 20°C. Thermal denaturation data were fit to standard equations by nonlinear least-squares regression using the Igor Pro (Wave Metrics, Inc.) assuming a two-state transition. All denaturation curves were fit to following equation:
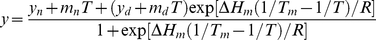
(1)where *y* represents the observed ellipticity; *y*_n_ and *m*_n_, *y*_d_ and *m*_d_ are the y-intercept and slope of the pre- and posttransitional baselines respectively; *T* is the temperature (in degrees Kelvin); *T*_m_ is the midpoint transition temperature; and Δ*H*_m_ is the enthalpy change for unfolding at *T*_m_ [27].

### Fluorescence perturbation assays

The affinities of SH3 domain variants for peptide VSL12 were measured by fluorescence perturbation assays, as described previously [28]. Aliquots of peptide solution were added to solutions of SH3 domain (0.5 µM) in PBS (Phosphate-buffered saline, pH 7.4). The mixture was incubated for 10 min at 20°C and then analyzed by a FP-777 fluorescence spectrophotometer (Jasco). The excitation wavelength was 278 nm (10 nm slit), and the emission wavelength was 350 nm (5 nm slit) for all experiments.

## Supporting Information

Figure S1 **ELISA of SH3(YNN)_28_ libraries at each round of selection.** The total amount of SH3(YNN)_28_ library that bound to the peptide immobilized (gray bars) and non-immobilized well (black bars) before (0) and after 1–5 rounds of mRNA-display selection were quantified by ELISA. Consequently, after 5 rounds of selection, the translated products of SH3(YNN)_28_ library non-specifically bound to ELISA plates, and no ligand-specific binder was enriched. Because the sequences of the non-specific binders contain a partial frameshift in the fixed region in the SH3 gene (data not shown), the non-specific binders might have no SH3-like structure. Further, their sequences contain a lot of basic amino acids (data not shown), suggesting that they would probably bind to carboxylic acid group on the surface of the affinity beads and the ELISA plates. Such non-specific binders might also be included in the initial SH3(RNN)_28_ and SH3(NNN)_28_ libraries, but not be observed after selection probably due to the competition with a lot of specific-binders in the libraries.
(TIFF)

Figure S2 **Purification of proteins selected from the SH3(RNN)_28_ and SH3(NNN)_28_ libraries.** The selected proteins with His_6_ tag, were overexpressed in *E. coli*. The insoluble fractions of the crude lysate of selected proteins were purified on Ni-NTA resins. The samples before (N, non purified) and after purification (P, purified) were resolved by 16.5% Tricine sodium dodecyl sulfate-polyacrylamide gel electrophoresis and stained with Coomassie brilliant blue. The purified proteins (∼9 kDa) showed single bands.
(TIFF)

Figure S3 **Circular dichroism spectra of SH3 domains in folded and unfolded states.** The folded and unfolded samples were measured at 20°C and 99°C, respectively. (A) wild-type; (B) R13; (C) N17. Although the CD spectra of β-sheet proteins usually have minima at ∼217 nm, the folded SH3 domains have unusual maxima at 220 nm (solid line) that are thought to be caused by the environment of the aromatic residues or β-turn conformations [22]. Further, the CD spectra of the unfolded SH3 domains have unusual minima at 220 nm (broken line), probably due to the presence of non-native hydrophobic clusters organized by Trp rings within disordered states [29].
(TIFF)

Table S1 **Percentage of each amino acid in the randomized region of the initial and third rounds of libraries.**
(DOC)

Table S2 **Oligonucleotide sequences used in this study.**
(DOC)

Table S3 **Percentage of each nucleotide at each position of the designed codons.**
(DOC)
