# Supplementary figures and images for: Comparison of the Frequency of Functional SH3 Domains with Different Limited Sets of Amino Acids Using mRNA Display
Source: PLoS One. 2011 Mar 21;6(3):e18034. doi: 10.1371/journal.pone.0018034 (PMC3061877; doi:10.1371/journal.pone.0018034)

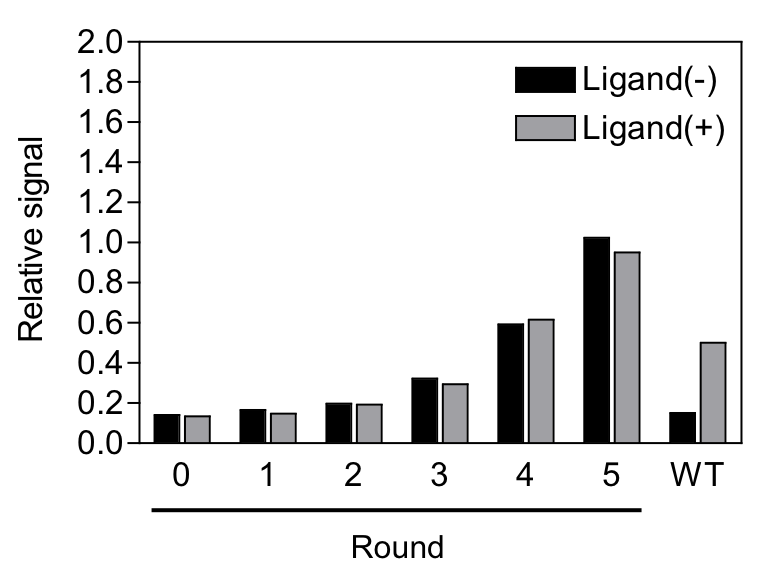

Supplement: Figure S1 — ELISA of SH3(YNN)28 libraries at each round of selection. The total amount of SH3(YNN)28 library that bound to the peptide immobilized (gray bars) and non-immobilized well (black bars) before (0) and after 1–5 rounds of mRNA-display selection were quantified by ELISA. Consequently, after 5 rounds of selection, the translated products of SH3(YNN)28 library non-specifically bound to ELISA plates, and no ligand-specific binder was enriched. Because the sequences of the non-specific binders contain a partial frameshift in the fixed region in the SH3 gene (data not shown), the non-specific binders might have no SH3-like structure. Further, their sequences contain a lot of basic amino acids (data not shown), suggesting that they would probably bind to carboxylic acid group on the surface of the affinity beads and the ELISA plates. Such non-specific binders might also be included in the initial SH3(RNN)28 and SH3(NNN)28 libraries, but not be observed after selection probably due to the competition with a lot of specific-binders in the libraries. (TIFF) [file pone.0018034.s001.tiff]

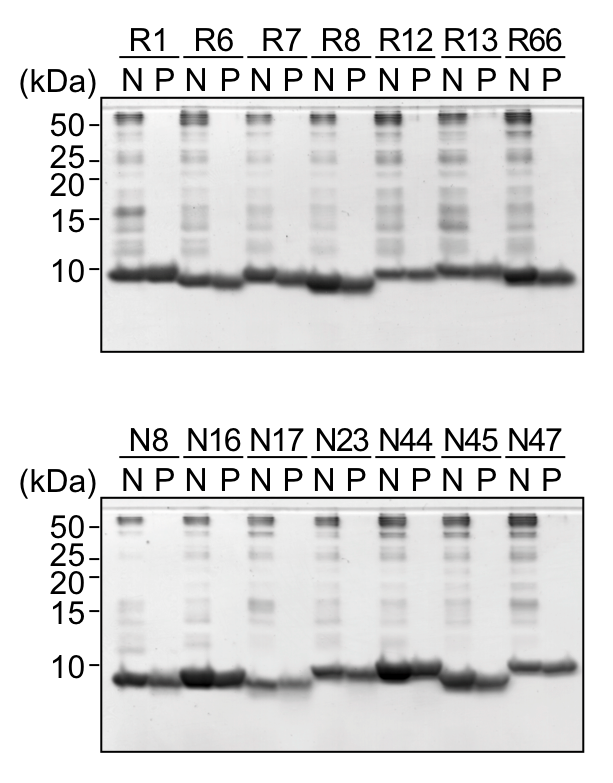

Supplement: Figure S2 — Purification of proteins selected from the SH3(RNN)28 and SH3(NNN)28 libraries. The selected proteins with His6 tag, were overexpressed in E. coli. The insoluble fractions of the crude lysate of selected proteins were purified on Ni-NTA resins. The samples before (N, non purified) and after purification (P, purified) were resolved by 16.5% Tricine sodium dodecyl sulfate-polyacrylamide gel electrophoresis and stained with Coomassie brilliant blue. The purified proteins (∼9 kDa) showed single bands. (TIFF) [file pone.0018034.s002.tiff]

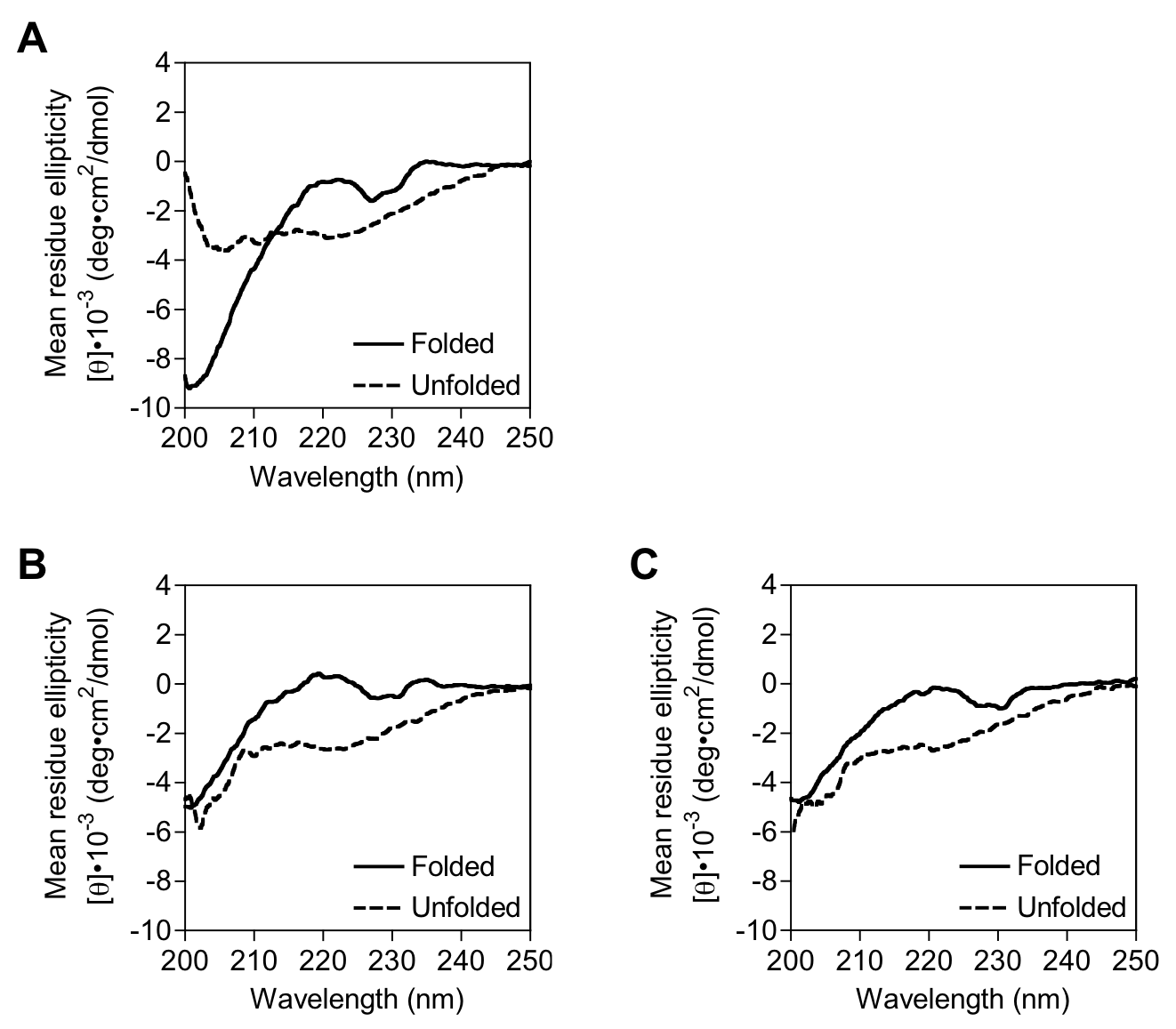

Supplement: Figure S3 — Circular dichroism spectra of SH3 domains in folded and unfolded states. The folded and unfolded samples were measured at 20°C and 99°C, respectively. (A) wild-type; (B) R13; (C) N17. Although the CD spectra of β-sheet proteins usually have minima at ∼217 nm, the folded SH3 domains have unusual maxima at 220 nm (solid line) that are thought to be caused by the environment of the aromatic residues or β-turn conformations [22]. Further, the CD spectra of the unfolded SH3 domains have unusual minima at 220 nm (broken line), probably due to the presence of non-native hydrophobic clusters organized by Trp rings within disordered states [29]. (TIFF) [file pone.0018034.s003.tiff]
