# Supplementary material for: Comparison of the Frequency of Functional SH3 Domains with Different Limited Sets of Amino Acids Using mRNA Display
Source: PLoS One. 2011 Mar 21;6(3):e18034. doi: 10.1371/journal.pone.0018034 (PMC3061877; doi:10.1371/journal.pone.0018034)
Supplement: Table S1 — Percentage of each amino acid in the randomized region of the initial and third rounds of libraries. (DOC) [file pone.0018034.s004.doc]

**Table S1**. Percentage of each amino acid in the randomized region of the initial and third rounds of libraries

| Amino acids | Wild-type | SH3(NNN)_28_ | | SH3(RNN)_28_ | | SH3(YNN)_28_ |
| --- | --- | --- | --- | --- | --- | --- |
|  |  | 0 round^a^ | 3 round^b^ | 0 round^a^ | 3 round^b^ | 0 round^a^ |
| Gly | 3.6 | 5.7 | 3.8 | 9.2 | 2.9 | 0.0 |
| Ala | 3.6 | 4.6 | 4.6 | 7.5 | 5.0 | 0.0 |
| Val | 7.1 | 7.8 | 10.7 | 12.8 | 16.4 | 0.0 |
| Leu | 7.1 | 8.0 | 9.7 | 0.0 | 0.0 | 21.1 |
| Ile | 0.0 | 5.2 | 6.9 | 8.3 | 13.2 | 0.0 |
| Met | 0.0 | 5.0 | 5.3 | 8.0 | 7.1 | 0.0 |
| Pro | 3.6 | 3.1 | 2.7 | 0.0 | 0.0 | 8.0 |
| Phe | 3.6 | 3.0 | 2.1 | 0.0 | 0.0 | 7.8 |
| Trp | 0.0 | 2.1 | 1.9 | 0.0 | 0.0 | 5.5 |
| Ser | 10.7 | 7.1 | 6.9 | 6.0 | 7.4 | 9.0 |
| Thr | 17.9 | 6.0 | 5.5 | 9.5 | 12.0 | 0.0 |
| Asn | 7.1 | 5.9 | 6.7 | 9.4 | 7.8 | 0.0 |
| Gln | 3.6 | 2.9 | 2.7 | 0.0 | 0.0 | 7.6 |
| Tyr | 0.0 | 3.4 | 3.2 | 0.0 | 0.0 | 8.9 |
| Cys | 0.0 | 2.1 | 0.8 | 0.0 | 0.0 | 5.7 |
| Lys | 7.1 | 5.7 | 6.5 | 9.1 | 10.7 | 0.0 |
| Arg | 10.7 | 7.4 | 5.9 | 5.7 | 4.4 | 9.9 |
| His | 3.6 | 3.0 | 2.3 | 0.0 | 0.0 | 7.9 |
| Asp | 3.6 | 4.5 | 4.4 | 7.4 | 5.9 | 0.0 |
| Glu | 7.1 | 4.4 | 7.1 | 7.1 | 7.1 | 0.0 |

^a^ Calculated based on nucleotide distribution at each position of the designed codons.

^b^ Calculated from 17 kinds of functional sequences from the third round of SH3(NNN)_28_, SH3(RNN)_28_.
