# Supplementary material for: Comparison of the Frequency of Functional SH3 Domains with Different Limited Sets of Amino Acids Using mRNA Display
Source: PLoS One. 2011 Mar 21;6(3):e18034. doi: 10.1371/journal.pone.0018034 (PMC3061877; doi:10.1371/journal.pone.0018034)
Supplement: Table S2 — Oligonucleotide sequences used in this study. (DOC) [file pone.0018034.s005.doc]

**Table S2**. Oligonucleotide sequences used in this study

| Name | Sequence (5’ to 3’)^a^ |
| --- | --- |
| Fragment 1 | GAAGCTCAGGTCCGTCTCzyxzyxGCTzyxATAATCGTAzyxGGCzyxzyxzyxAGATCTCGCATCCCCCATCTGCTGGCCGCCTGTCATGGACGCCAT |
| Fragment 2 | GAGACGGACCTGAGCTTCXYZXYZGGCGAAXYZCTGXYZATCXYZXYZXYZXYZXYZGGCGACTGGTGGCTCGCG |
| Fragment 3 | GACGTAGTTGCTCGGAATGTACCCzyxzyxzyxzyxzyxzyxzyxzyxCGCGAGCCACCAGTCGCC |
| Fragment 4 | GGGTACATTCCGAGCAACTACGTCXYZXYZXYZXYZCTCGAGAGGGGCGATTACAAAGACGACGACGATAAGAAAAAAAA |
| SPO7tagF-mut2 | ATTTAGGTGACACTATAGAACAACAACAACAACAAACAACAACAAAATGGCGTCCATGACAGGCGGCCAGCAGATGGGGGATGCG |
| FLAG1A-mut2 | TTTTTTTTCTTATCGTCGTCGTCTTTGTAATCGCCCCTCTCGAG |
| N8-F | CTGAGGATCTATGATGACTTCAGT |
| N8-R | TCTCGAGGTTATACATGGC |
| N16-F | GATCCTCAATTCCAGCCTG |
| N16-R | CTCTCGAGCGCGTAATACTT |
| R2-F | CTGACTATCATCGATAACATCAGT |
| R2-R | TCTCGAGAGTCTTGCTCAC |
| R6-F | GGATGCGAGATCTACTGTTGTT |
| R6-R | TATCACCCGTCATCCTCTTCAC |

^a^ The compositions of mixed-bases X, Y, Z, x, y and z in Fragments 1 to 4 are shown in Table S3.
