# Supplementary material for: Comparison of the Frequency of Functional SH3 Domains with Different Limited Sets of Amino Acids Using mRNA Display
Source: PLoS One. 2011 Mar 21;6(3):e18034. doi: 10.1371/journal.pone.0018034 (PMC3061877; doi:10.1371/journal.pone.0018034)
Supplement: Table S3 — Percentage of each nucleotide at each position of the designed codons. (DOC) [file pone.0018034.s006.doc]

**Table S3**. Percentage of each nucleotide at each position of the designed codons

| Library | Position^a^ | %T | %C | %A | %G |
| --- | --- | --- | --- | --- | --- |
| SH3(NNN)_28_ | X | 20 | 18 | 35 | 27 |
|  | Y | 29 | 17 | 33 | 21 |
|  | Z | 22 | 29 | 0 | 49 |
|  | x | 35 | 27 | 20 | 18 |
|  | y | 33 | 21 | 29 | 17 |
|  | z | 0 | 49 | 22 | 29 |
| SH3(RNN)_28_ | X | 0 | 0 | 56 | 44 |
|  | Y | 29 | 17 | 33 | 21 |
|  | Z | 22 | 29 | 0 | 49 |
|  | x | 56 | 44 | 0 | 0 |
|  | y | 33 | 21 | 29 | 17 |
|  | z | 0 | 49 | 22 | 29 |
| SH3(YNN)_28_ | X | 53 | 47 | 0 | 0 |
|  | Y | 29 | 17 | 33 | 21 |
|  | Z | 22 | 29 | 0 | 49 |
|  | x | 0 | 0 | 53 | 47 |
|  | y | 33 | 21 | 29 | 17 |
|  | z | 0 | 49 | 22 | 29 |

^a^ The positions (X, Y, Z, x, y and z) are shown in Table S2.
